# Peer review of "Phenotypical, Behavioral, and Systemic Hallmarks in End-Point Mouse Scenarios"

_animals, 2025, doi:10.3390/ani15040521_

Round 1

Reviewer 1 Report

Comments and Suggestions for Authors

The manuscript presents careful comparison of phenotypical, behavioral, and systemic features used in evaluating aging mice.  Comparison includes adult, aged, and aged-frail mice.  The data shared will be useful to investigators in the field and to reviewers assessing the welfare of animals used in this type of research.  

Line 52 – prefer euthanized to “sacrificed”

Line 132: How were the animals euthanized?

Lines 132-133 – suggest reorganizing sentence –

 One hour after behavioural evaluation, the animals were euthanized and necropsied.  The liver, spleen, WAT, and triceps surae muscle were weighed and preserved for future analysis.

Line 140-142 – not clear if this final sentence of the paragraph is redundant or referencing organs and tissues aside from liver, spleen, WAT and triceps surae muscle, perhaps?

Line 264:  Although “not justified by muscular loss” , could increased latency to rearing in the mice reflect musculoskeletal pain?

Line 396: prefer euthanized to “sacrificed”

Author Response

The manuscript presents careful comparison of phenotypical, behavioral, and systemic features used in evaluating aging mice.  Comparison includes adult, aged, and aged-frail mice.  The data shared will be useful to investigators in the field and to reviewers assessing the welfare of animals used in this type of research. 
Comment 1: Line 52 – prefer euthanized to “sacrificed”.
Line 132: How were the animals euthanized?
Lines 132-133 – suggest reorganizing sentence –
Response: We thank the reviewer for his comments. We have included the reviewer's recommendation. The Lines 52, 132-133 have been modified.
Comment 2: One hour after behavioural evaluation, the animals were euthanized and necropsied.  The liver, spleen, WAT, and triceps surae muscle were weighed and preserved for future analysis. 
Line 140-142 – not clear if this final sentence of the paragraph is redundant or referencing organs and tissues aside from liver, spleen, WAT and triceps surae muscle, perhaps?
Response: We have included the reviewer's recommendation. The Lines 140-142 have been modified.
Line 264:  Although “not justified by muscular loss” , could increased latency to rearing in the mice reflect musculoskeletal pain?
Line 396: prefer euthanized to “sacrificed”
Response: We have included the reviewer's recommendation. The Lines 140-142, 264, 396, 441-443 have been modified.

Reviewer 2 Report

Comments and Suggestions for Authors

Title: Phenotypical, behavioural and systemic hallmarks in end-point mice scenarios

Authors: Lidia Castillo-Mariqueoet al.

Journal: Animals-3414322

The purpose of this paper is to examine the behavioural markers that predict imminent death in old C57BL/6J mice.  To do this, 16-month-old male mice that were classified as "end-point" were compared with 16 month old mice that were "healthy" and both of these groups were compared with a group of 6 month old mice. The aim was to define a behavioural phenotype for end of life, which could help to identify mice that were on their last legs and should be euthanized.

If you are doing aging research with mouse models, this is an important question: can you predict when a mouse will die, based on its behavioural profile.

The Introduction cites many papers on age-related injuries, deterioration and diseases and uses these to select a set of behaviours to score. These include frailty, homecage behaviour, and appearance, which are then correlated with physical measures, including muscle weight, spleen, liver, and adipose tissue weights as well as body weight.

Mice were divided into "end-point" and "normal aging", but there is no indication of how this was done.

Only male mice were used.  Do male and female C57BL/6J mice age at the same rate or are there sex differences in the rate of aging?

There is no definition of how the two groups were defined. How did you determine if a particular 16-month-old mouse was in the "normal aging" or the "end point" group? Two references are cited on lines 109 & 110, but there are no decision criteria given.

C57BL/6J mice live up to 3 years of age with the expected life span (50% mortality) at 890 days (29.6 months). The mice in this study were examined at 16 months = 480 days, which does not even put them into the "old-age" range (540-694 days) [See Rae et al. 2015. The problem of genotype and sex differences in life expectancy in transgenic AD mice.  Neuroscience and Biobehavioural Reviews, 57, 238-251.]

Mice were euthanized after the behavioural tests at 16 months of age and so never reached old age or the mean lifespan of 890 days.

Behavioural measures were then correlated with anatomical measures in these relatively young mice.

Some of the measures taken met the criterion for parametric analysis (weights) while others were on rating scales so non-parametric tests should be done.

Since the sample sizes were very small the data would not meet the criteria for parametric tests such as the t-test and U-tests should be done.

As for the correlations, non-parametric Spearman or Kendall correlations should be done.

Also there are a large number of correlations and some could be spurious. How did you correct for multiple correlations?

As presented in Tables, the numbers are covered with coloured panels and are hard to read, especially when covered in blue. 

Results

The behaviours in the "physical frailty phenotype checklist " are not defined clearly. They were scored 0 or 1, which is nominal data. Chi square and Fisher tests were not listed in the section on statistical analyses.

In the behavioural tests only latency was measured in the "corner test" [What exactly is the corner test? It seems to be used for sensori-motor dysfunction after stroke. Why is it used here?  [see Zhang et al. 2002. A test for detecting long-term sensorimotor dysfunction in the mouse after focal cerebral ischemia. Journal of Neuroscience Methods 117, 207-214]

Latency to rear is a weak measure of motor ability. Number of rears in a given time period is a measure that shows age-related decline. [See O'Leary et al., 2020. Age-related deterioration of motor function in male and female 5xFAD mice from 3-16 months of age. Genes, Brain and Behavior, 19:e12538.  https://doi.org/10.1111/gbb.12538]. In that study, 15-16 month old C57BL/6J x SJL mice of both sexes showed very little decline in motor behaviour, but 5xFAD mice showed a significant age-related decline.

C57BL6J mice can perform well in behavioural tests up to 24 months of age [See Brust V. et al. 2015. Lifetime development of behavioural phenotype in the house mouse. Frontiers in Zoology, 12(Suppl 1): S17.]

NMRI mice have been tested between 17 and 22 months of age [See Lamberty & Gower. 1992. Age-related changes in spontaneous behaviour and learning in NMRI mice from middle to old age. Physiology and behaviour, 51, 81-88].

Likewise, Yanai and Endo tested male C57BL/6J mice up to 22 months of age to produce a behavioural aging profile [see Yanai S and Endo S (2021) Functional Aging in Male C57BL/6J Mice Across the Life-Span: A Systematic Behavioral Analysis of Motor, Emotional, and Memory Function to Define an Aging Phenotype. Front. Aging Neurosci. 13:697621. doi: 10.3389/fnagi.2021.697621].

Braz et al (2024) tested the responses of male and female C57BL/6 mice to mechanical stimulation up to 24 months of age [see Braz JM, et al. 2024. Pain and itch processing in aged mice.The Journal of Pain, 25, 53-63.]

Finally, Ederer M-L., et al.  (2022) tested C57BL/6J mice up ton28 months of age in the Barnes maze.  [see Ederer M-L., et al. 2022. Voluntary Wheel Running in Old

C57BL/6 Mice Reduces Age-Related Inflammation in the Colon but Not in the Brain. Cells 11, 566. https://doi.org/10.3390/cells11030566]

The problem with the present paper is that mice were not followed until their natural end of life but were arbitrarily euthanized at 16 months of age.  Thus, we have no idea of how long these mice could have lived, and of the individual differences in measures of frailty as they aged.

Figure 2C is off of the page.

What improvements need to be made?

1. Increase the sample sizes

2. Use females as well as males.

3. Examine mice up to 30 months of age

4. Determine the natural age of death, rather than euthanizing them all at 16 months of

            age

5. Show the data used to divide the 16 month-old mice into "end-point" and "normal         aging" groups.

6. Show frequencies of movement, rearing and exploratory activity over 5-10 min behavioural tests not just latencies.

7. Test mice more than once

8. Use non-parametric statistical tests for small samples

9. Make sure all of the figures are on the page

10. Do not use coloured panels to obscure the numbers in Table 1c.

11. Define all of the measures in the methods section.

12. Improve the writing. There are many errors in English grammar. Some sentences      are unclear. For example:

            lines 28-31 of the abstract

            lines 38-41 of the Introduction

            lines 43-44 of Introduction

            and others

13. Show a checklist of "physical frailty genotype " behaviours and describe how each     was scored.

14. The definition of the two types of kyphosis is unclear. How were they differentiated?

15. Describe the corner test and how it was used. It seems to be a test for a stroke          model.

16. The section on statistics does not list all of the statistics used.

17. There are behaviours in the results that are not listed in the methods (defecation,      urination).

18. The correlation tables make no sense to me.  How can kyphosis of 6-month-old mice be correlated with anything if all of the scores were zero? The same for piloerection.

This sort of study would be useful if it provided data on age-related changes that predicted end of life and gave researchers a set of criteria for determining when to euthanize old mice, but the paper does not do that. The paper by Brust V. et al. [2015. Lifetime development of behavioural phenotype in the house mouse. Frontiers in Zoology, 12(Suppl 1): S17.] notes the need for such studies.

Comments on the Quality of English Language

see attached letter

Author Response

Reviewer 2:
The purpose of this paper is to examine the behavioural markers that predict imminent death in old C57BL/6J mice.  To do this, 16-month-old male mice that were classified as "end-point" were compared with 16 month old mice that were "healthy" and both of these groups were compared with a group of 6 month old mice. The aim was to define a behavioural phenotype for end of life, which could help to identify mice that were on their last legs and should be euthanized.
Comment 1: If you are doing aging research with mouse models, this is an important question: can you predict when a mouse will die, based on its behavioural profile.
Response: We thank the reviewer for his comments. We have a previous paper related to sepsis where we show to which extend the phenotype is a kind of predictor of their survival during ‘sickness behavior’: Giménez-Llort L, Ramírez-Boix P, de la Fuente M. Mortality of septic old and adult male mice correlates with individual differences in premorbid behavioral phenotype and acute-phase sickness behavior. Exp Gerontol. 2019 Nov;127:110717. doi: 10.1016/j.exger.2019.110717. Epub 2019 Aug 31. PMID: 31479727.
Comment 2: The Introduction cites many papers on age-related injuries, deterioration and diseases and uses these to select a set of behaviours to score. These include frailty, homecage behaviour, and appearance, which are then correlated with physical measures, including muscle weight, spleen, liver, and adipose tissue weights as well as body weight. Mice were divided into "end-point" and "normal aging", but there is no indication of how this was done. Only male mice were used.  Do male and female C57BL/6J mice age at the same rate or are there sex differences in the rate of aging? 
Response: We have included the reviewer's recommendation. The Lines 435-440 have been modified.
Comment 3: There is no definition of how the two groups were defined. How did you determine if a particular 16-month-old mouse was in the "normal aging" or the "end point" group? Two references are cited on lines 109 & 110, but there are no decision criteria given.
Response: We have included the reviewer's recommendation. The Lines 114-116 have been modified.
Comment 4: C57BL/6J mice live up to 3 years of age with the expected life span (50% mortality) at 890 days (29.6 months). The mice in this study were examined at 16 months = 480 days, which does not even put them into the "old-age" range (540-694 days) [See Rae et al. 2015. The problem of genotype and sex differences in life expectancy in transgenic AD mice.  Neuroscience and Biobehavioural Reviews, 57, 238-251.]
Response: According to our survival curves, as depicted in most of our work on aging, the age of 16 or 18 are the one that express aging profiles at psico-neuroimmunoendocrine network level (literature in collaboration with De la Fuente, M. et al.). Also at this age, is when most veterinarian ‘end-of-life’ red labels are common in the animal department. 
Comment 5: Mice were euthanized after the behavioural tests at 16 months of age and so never reached old age or the mean lifespan of 890 days.
Response: According to our psico-neuroimmunoendocrine network, the age of 16 already presents behavioral and neuroimmune profiles of 18th month of age. As mentioned, our age frames depend on the ‘end-of-life’ red flags.
Comment 6: Behavioural measures were then correlated with anatomical measures in these relatively young mice. Some of the measures taken met the criterion for parametric analysis (weights) while others were on rating scales so non-parametric tests should be done. Since the sample sizes were very small the data would not meet the criteria for parametric tests such as the t-test and U-tests should be done. As for the correlations, non-parametric Spearman or Kendall correlations should be done.
Response: We have included the reviewer's recommendation. Lines 163-166 have been modified.
Comment 7: Also there are a large number of correlations and some could be spurious. How did you correct for multiple correlations?
Response: We appreciate the referee’s concern regarding the potential for spurious associations due to multiple comparisons. However, in our study, each linear regression analysis was conducted independently, with variables chosen based on established biological relevance rather than an exploratory approach. Thus, applying a multiple testing correction in this context would not be appropriate, as it assumes that all models are part of a single family of comparisons, which is not the case in our approach. Instead, we relied on biological relevance and consistency with prior research to ensure the robustness of our findings.
If needed, we can clarify this further in the manuscript to ensure transparency in our statistical methodology.
Comment 8: As presented in Tables, the numbers are covered with coloured panels and are hard to read, especially when covered in blue. 
Response: We have corrected the table 1 colours.
Comment 9: Results. The behaviours in the "physical frailty phenotype checklist " are not defined clearly. They were scored 0 or 1, which is nominal data. Chi square and Fisher tests were not listed in the section on statistical analyses.
Response: We have included the reviewer's recommendation. Lines 127-133; 167-169 have been modified.
Comment 9: In the behavioural tests only latency was measured in the "corner test" [What exactly is the corner test? It seems to be used for sensori-motor dysfunction after stroke. Why is it used here?  [see Zhang et al. 2002. A test for detecting long-term sensorimotor dysfunction in the mouse after focal cerebral ischemia. Journal of Neuroscience Methods 117, 207-214]
Response: The test is an adaptation of ‘corner test’ (two walls in the shape of a corner, Schallert et al., 82, 83; adapted later by Zhang et al., 2002 to assess sensory-motor function) by Giménez-Llort et al., as a ‘neophobia test’ (no sensory-motor deficits are ensured by concurrent sensorimotor test assessment with other tests) after observing that during the period of transfer inside a cage from animal room to test rooms for assessment of animals in the open-field, the mice perform a short exploratory activity that implies visiting the corners followed by rearing. This behavior was predictive of observations in the subsequent open field test and was considered the most pure record of neophobia, since the transfer from the homecage to the ‘transfer cage’ was the first ‘exposure’ to a novel environment that the animals confronted on that ‘testing day’. The method and results were included for the first time in the book Giménez-Llort et al., 2006, prior to the review Giménez-Llort et al., 2007 and the first research original paper Giménez-Llort et al., 2010. Since then, many authors have used it to assess neophobia or response to novelty in a fast and easy manner. In the reference provided by the reviewer, the protocol is the original version for sensorymotor function (which resembles the cylinder test we have also used in one of our studies on hypoxic isquemic injury Muntsant A, Shrivastava K, Recasens M, Giménez-Llort L. Severe Perinatal Hypoxic-Ischemic Brain Injury Induces Long-Term Sensorimotor Deficits, Anxiety-Like Behaviors and Cognitive Impairment in a Sex-, Age- and Task-Selective Manner in C57BL/6 Mice but Can Be Modulated by Neonatal Handling. Front Behav Neurosci. 2019 Feb 13;13:7. doi: 10.3389/fnbeh.2019.00007. PMID: 30814939; PMCID: PMC6381068.) Therefore, to avoid the potential confounding factor, we started to refer to Corner test for neophobia.
The references for corner test for sensorymotor function:
[Zhang et al., 2002; Corner test: in the home cage, a mouse was placed between two boards each with dimension of 30/ 20/1 cm3 . The edges of the two boards were attached at a 308 angle with a small opening along the joint between the two boards to encourage entry into the corner. The mouse was placed between the two angled boards facing the corner and half way to the corner. When entering deep into the corner both sides of the vibrissae are stimulated together. The mouse then rears forward and upward, then turns back to face the open end. The non-ischemic mouse turns either left or right, but the ischemic mouse preferentially turns toward the non-impaired, ipsilateral (right) side. The turns in one versus the other direction were recorded from ten trials for each test. Turning movements that were not part of a rearing movement were not scored.)
Schallert T, Upchurch M, Lobaugh N, Farrar SB, Spirduso WW, Gilliam P, et al. Tactile extinction: distinguishing between sensorimotor and motor asymmetries in rats with unilateral nigrostriatal damage. Pharmacol Biochem Behav. 1982;16:455–462. doi: 10.1016/0091-3057(82)90452-x.
Schallert T, Upchurch M, Wilcox RE, Vaughn DM. Posture-independent sensorimotor analysis of inter-hemispheric receptor asymmetries in neostriatum. Pharmacol Biochem Behav. 1983;18:753–759. doi: 10.1016/0091-3057(83)90019-9.
A short paragraph on this regard, has been added to clarify this issue.
Comment 10: Latency to rear is a weak measure of motor ability. Number of rears in a given time period is a measure that shows age-related decline. [See O'Leary et al., 2020. Age-related deterioration of motor function in male and female 5xFAD mice from 3-16 months of age. Genes, Brain and Behavior, 19:e12538.  https://doi.org/10.1111/gbb.12538]. In that study, 15-16 month old C57BL/6J x SJL mice of both sexes showed very little decline in motor behaviour, but 5xFAD mice showed a significant age-related decline.
C57BL6J mice can perform well in behavioural tests up to 24 months of age [See Brust V. et al. 2015. Lifetime development of behavioural phenotype in the house mouse. Frontiers in Zoology, 12(Suppl 1): S17.]
NMRI mice have been tested between 17 and 22 months of age [See Lamberty & Gower. 1992. Age-related changes in spontaneous behaviour and learning in NMRI mice from middle to old age. Physiology and behaviour, 51, 81-88].
Likewise, Yanai and Endo tested male C57BL/6J mice up to 22 months of age to produce a behavioural aging profile [see Yanai S and Endo S (2021) Functional Aging in Male C57BL/6J Mice Across the Life-Span: A Systematic Behavioral Analysis of Motor, Emotional, and Memory Function to Define an Aging Phenotype. Front. Aging Neurosci. 13:697621. doi: 10.3389/fnagi.2021.697621].
Braz et al (2024) tested the responses of male and female C57BL/6 mice to mechanical stimulation up to 24 months of age [see Braz JM, et al. 2024. Pain and itch processing in aged mice.The Journal of Pain, 25, 53-63.]
Finally, Ederer M-L., et al.  (2022) tested C57BL/6J mice up ton28 months of age in the Barnes maze.  [see Ederer M-L., et al. 2022. Voluntary Wheel Running in Old
C57BL/6 Mice Reduces Age-Related Inflammation in the Colon but Not in the Brain. Cells 11, 566. https://doi.org/10.3390/cells11030566]
Response: We appreciate your recommendations. In previous reports from this animal colony, we detected a motor behavior distinguishing 3xTg-AD mice from their controls. In males, we have detected that postural alterations limit exploratory activity, correlating with gait and vertical and horizontal activity. These changes have been evidenced from an early age. When, for example, structural kyphosis is present, the limitation is more severe. On the other hand, we have previously reported that the functional decline between males and females is very heterogeneous. Females usually present better motor performance but with a higher level of deterioration in frailty, so studying males and females is complex due to the number of factors present. 
Castillo-Mariqueo, L., & Giménez-Llort, L. (2021a). Translational Modeling of Psychomotor Function in Normal and AD-Pathological Aging With Special Concerns on the Effects of Social Isolation. Frontiers in Aging, 2, 648567. https://doi.org/10.3389/fragi.2021.648567 Castillo-Mariqueo, L., & Giménez-Llort, L. (2021b). Kyphosis and bizarre patterns impair spontaneous gait performance in end-of-life mice with Alzheimer’s disease pathology while gait is preserved in normal aging. Neuroscience Letters, 136280. https://doi.org/10.1016/J.NEULET.2021.136280. Castillo-Mariqueo, L., Giménez-Llort, L. (2019). Humans to Mouse Models. In Gerontology And Geriatric Research (Vol. 2, Issue 2). Castillo-Mariqueo, L., Pérez-García, M. J., & Giménez-Llort, L. (2021a). Modeling Functional Limitations, Gait Impairments, and Muscle Pathology in Alzheimer’s Disease: Studies in the 3xTg-AD Mice. Biomedicines, 9(10). https://doi.org/10.3390/BIOMEDICINES9101365. Castillo Mariqueo, L., Alveal-Mellado, D., & Gimenez-LLort, L. (2021b). Hindlimb clasping, kyphosis and piloerection: Frailty markers from middle to very old ages in mice. In EUROPEAN JOURNAL OF NEUROLOGY (Vol. 28, pp. 447-447). 111 RIVER ST, HOBOKEN 07030-5774, NJ USA: WILEY.
Per your suggestions, we have included modifications in the paragraphs you indicated.
Comment 11: The problem with the present paper is that mice were not followed until their natural end of life but were arbitrarily euthanized at 16 months of age.  Thus, we have no idea of how long these mice could have lived, and of the individual differences in measures of frailty as they aged.
Response: We have included the reviewer's recommendation. The Lines 115-136 have been modified.
Comment 12: Figure 2C is off of the page. What improvements need to be made?
1. Increase the sample sizes
2. Use females as well as males.
3. Examine mice up to 30 months of age
4. Determine the natural age of death, rather than euthanizing them all at 16 months of age
5. Show the data used to divide the 16 month-old mice into "end-point" and "normal  aging" groups.
6. Show frequencies of movement, rearing and exploratory activity over 5-10 min behavioural tests not just latencies.
7. Test mice more than once
8. Use non-parametric statistical tests for small samples
9. Make sure all of the figures are on the page
10. Do not use coloured panels to obscure the numbers in Table 1c.
11. Define all of the measures in the methods section.
Response: We have included the reviewer's recommendation. Lines 124-147, 314-315, 371-374, 382-383, 391-394, 428-435.
Comment 13: Improve the writing. There are many errors in English grammar. Some sentences      are unclear. For example:
            lines 28-31 of the abstract
            lines 38-41 of the Introduction
            lines 43-44 of Introduction
            and others
Response: Response: We have included the reviewer's recommendation
Comment 14: Show a checklist of "physical frailty genotype " behaviours and describe how each  was scored.
The definition of the two types of kyphosis is unclear. How were they differentiated?
Describe the corner test and how it was used. It seems to be a test for a stroke model.
The section on statistics does not list all of the statistics used.
There are behaviours in the results that are not listed in the methods (defecation, urination).
The correlation tables make no sense to me.  How can kyphosis of 6-month-old mice be correlated with anything if all of the scores were zero? The same for piloerection.
This sort of study would be useful if it provided data on age-related changes that predicted end of life and gave researchers a set of criteria for determining when to euthanize old mice, but the paper does not do that. The paper by Brust V. et al. [2015. Lifetime development of behavioural phenotype in the house mouse. Frontiers in Zoology, 12(Suppl 1): S17.] notes the need for such studies.
Response: We have included the reviewer's recommendation. Lines 124-147, 457-467.

Reviewer 3 Report

Comments and Suggestions for Authors

This work consists of the analysis of the physical and behavioural hallmarks of a set of 16 month-old mice at their end-point compared to age-matched counterparts with normal aging.

You have 2 “and” when stating the authors. A lot of typos, overall. Please change this.

Explain how the sample size was decided. Provide details of any a priori sample size calculation, if done. Describe any criteria used for including these animals.

“Before euthanasia, the animals were evaluated with a brief behavioural assessment”; Please expand this. “A score of 0 was assigned for normal aspects or 1 for abnormal aspects.” Was the score of 1 assigned only in the presence of all listed physical signs, or could it also be given for the presence of just some of them? Additionally, were intermediate conditions or partial manifestations considered, and how were they documented?

Figure 2 is not clearly visible. Please change this.

Please add some limitations to your study.

Author Response

Reviewer :
This work consists of the analysis of the physical and behavioural hallmarks of a set of 16-month-old mice at their end-point compared to age-matched counterparts with normal aging.
Comment 1: You have 2 “and” when stating the authors. A lot of typos, overall. Please change this.
Response: We thank the reviewer for his comments. We have included the reviewer's recommendation.
Comment 2: Explain how the sample size was decided. Provide details of any a priori sample size calculation, if done. Describe any criteria used for including these animals.
Responses: We have included the reviewer's recommendation. The lines 106-109 have been modified.
Comment 3: “Before euthanasia, the animals were evaluated with a brief behavioural assessment." Please expand this. “A score of 0 was assigned for normal aspects or 1 for abnormal aspects.” Was the score of 1 assigned only in the presence of all listed physical signs, or could it also be given for the presence of just some of them? Additionally, were intermediate conditions or partial manifestations considered, and how were they documented?
Response: We have included the reviewer's recommendation. The lines 115-136 have been modified.
Comment 4: Figure 2 is not clearly visible. Please change this.
Response: We have included the reviewer's recommendation. 
Comment 5: Please add some limitations to your study.
Responses: We have included the reviewer's recommendation. The lines 435-440 have been modified.